# miR‐6076 targets BCL6 in SH‐SY5Y cells to regulate amyloid‐β‐induced neuronal damage

**DOI:** 10.1111/jcmm.17999

**Published:** 2023-10-17

**Authors:** Yujian Lin, Lei Zhang, Mengyue Gao, Zixin Tang, Xiang Cheng, Haoming Li, Jianbing Qin, Meiling Tian, Guohua Jin, Xinhua Zhang, Wen Li

**Affiliations:** ^1^ Department of Human Anatomy, Institute of Neurobiology Nantong University Nantong Jiangsu PR China; ^2^ Co‐Innovation Center of Neuroregeneration Nantong University Nantong Jiangsu PR China; ^3^ Key Laboratory of Neuroregeneration of Jiangsu Province and Ministry of Education Nantong Jiangsu PR China

**Keywords:** Alzheimer's disease, apoptosis, Aβ_1‐42_, BCL6, miR‐6076

## Abstract

Amyloid‐β_1‐42_ (Aβ_1‐42_) is strongly associated with Alzheimer's disease (AD). The aim of this study is to elucidate whether and how miR‐6076 participates in the modulation of amyloid‐β (Aβ)‐induced neuronal damage. To construct the neuronal damage model, SH‐SY5Y cells were treated with Aβ_1‐42_. By qRT‐PCR, we found that miR‐6076 is significantly upregulated in Aβ_1‐42_‐treated SH‐SY5Y cells. After miR‐6076 inhibition, p‐Tau and apoptosis levels were downregulated, and cell viability was increased. Through online bioinformatics analysis, we found that B‐cell lymphoma 6 (BCL6) was a directly target of miR‐6076 via dual‐luciferase reporter assay. BCL6 overexpression mediated the decrease in elevated p‐Tau levels and increased viability in SH‐SY5Y cells following Aβ1‐42 treatment. Our results suggest that down‐regulation of miR‐6076 could attenuate Aβ_1‐42_‐induced neuronal damage by targeting BCL6, which provided a possible target to pursue for prevention and treatment of Aβ‐induced neuronal damage in AD.

## 1 INTRODUCTION

Alzheimer's disease (AD), the commonest cause of dementia, is characterized by memory loss, cognitive and functional abilities decline.^1^, ^2^ This neuropathological condition is manifested by neurodegeneration, neural loss, accumulation of amyloid plaques and development of neurofibrillary tangles in the brain.^3^, ^4^ According to the amyloid cascade hypothesis, Aβ_1‐42_, a primary toxic and/or aggregation‐prone species, could induce neurotoxicity leading to tau phosphorylation and neurodegeneration.^5^, ^6^, ^7^

MicroRNAs (miRNAs) are a class of short, endogenously initiated non‐coding RNAs that modulate gene expression at the post‐transcriptional level via recognition of cognate sequences.^8^ It is becoming evident that miRNAs are playing significant roles in many biological functions, including developmental timing and host–pathogen interactions as well as cell differentiation, embryogenesis, proliferation, apoptosis and tumorigenesis.^9^

According to numerous studies, several miRNAs in blood, cerebrospinal fluid or brain serve as candidate biomarkers for AD.^10^ The literature indicated that compared with aged matched control, miR‐98‐5p, miR‐885‐5p, miR‐483‐3p, miR‐342‐3p, miR‐191‐5p and miR‐let‐7d‐5p displayed significantly different expression levels in the serum of patients with AD.^11^ Muller et al.^12^ detected differences in the expression of miR‐146a, miR‐29a and miR‐125b in the cerebrospinal fluid of AD patients compared to control.

Likewise, there are many microRNAs involved in the regulation of different AD‐related factors and pathways. Wang et al.^13^ indicated that miR‐107 contributed to β‐site amyloid precursor protein‐cleaving enzyme 1 (BACE1) posttranscriptional regulation, and modulated APP secretase activity. Geekiyanage and Chan^14^ identified that the loss of miR‐137, miR‐181c, miR‐9 and miR‐29a/b‐1 increased serine palmitoyltransferase and in turn Aβ levels, representing additional risk factors in AD.

In the present study, the role of miR‐6076 in the pathogenesis of AD was investigated. The biological functions and underlying mechanism of miR‐6076 were investigated by establishing in vitro cell model of AD using Aβ_1‐42_‐treated SH‐SY5Y cells. Our study may provide a possible strategy for the prevention and treatment of Aβ‐induced neuronal damage in AD.

## 2 MATERIALS AND METHODS

### 2.1 Cell culture and treatment

SH‐SY5Y cells were obtained from Shanghai Zhong Qiao Xin Zhou Biotechnology (Shanghai, China), and were cultured in Dulbecco's modified Eagle's medium (DMEM) supplemented with 10% fetal bovine serum (Gibco, Thermo Fisher Scientific, Waltham, MA, USA) in a CO_2_ incubator. Aβ_1‐42_ was purchased from Sigma‐Aldrich (St. Louis, MO, USA) for the construction of the AD cell model. The aggregation of Aβ_1‐42_ was diluted to a concentration of 200 μM and SH‐SY5Y cells were treated with different concentrations or at different time points.^15^

### 2.2 Cell transfection

The miRNA mimic targeting miR‐6076 (miR‐6076) and their corresponding negative controls (miR‐NC mimic/miR‐NC inhibitor) were obtained from RIBOBIO (Guangzhou, China). The intellectual property rights of the sequence belong to Ribo biology, which were asked to be classified. BCL6‐overexpressing (pcDNA3.1, GenePharma; NCBI Accession Gene ID: 604; https://www.ncbi.nlm.nih.gov/nuccore/NM_001130845.1). The BCL6 gene overexpression plasmid vector pcDNA3.1‐EGFP and negative control were transfected into SH‐SY5Y using Lipofectamine 3000 (Invitrogen, Carlsbad, CA, USA) according to the instructions of manufacturer.

### 2.3 CCK‐8 assay

The viability of SH‐SY5Y cells with or without Aβ_1‐42_ treatment was detected by CCK‐8 assay (10 μL; Yeasen, Shanghai, China). Briefly, SH‐SY5Y cells were treated with or without Aβ^1‐42^, and miR‐6076 or BCL6 transfection. After that, CCK‐8 solution was supplemented to the culture plate and co‐incubated with the cells for 2 h at 37°C. The optical density (OD) at 450 nm was measured.

### 2.4 Flow cytometry assay

The apoptosis of SH‐SY5Y cells with or without Aβ_1‐42_ treatment were detected by an PE Annexin V Apoptosis Dectection kit (BD Biosciences, San Jose, CA, USA). In brief, treated cells were harvested and resuspended with 1 × binding buffer. Then, PE Annexin V (5 μL) and 7‐AAD (5 μL) were added to the binding buffer and incubated for 15 min in the dark. The apoptotic rate was analysed by a FACScan® flow cytometry (BD Biosciences). Q1: Percentage of Necrotic cells; Q2: Percentage of late apoptotic cells; Q3: Percentage of Early apoptosis; Q4: Percentage of Cells without apoptosis. Apoptotic cells % were calculated by Q2 + Q3.

### 2.5 Quantitative real‐time polymerase chain reaction (qRT‐PCR)

Total RNA was extracted using TRIzol Reagent (Vazyme Biotech, Nanjing, China) following the manufacturer's protocol. Prime‐Script RT reagent Kit (Vazyme Biotech) or miRcute miRNA First‐Strand cDNA Synthesis Kit (TIANGEN BIOTECH, Beijing, China) was used to generate cDNA. The SYBR Green (Roche, Basel, Switzerland) or miRcute miRNA qPCR Detection Kit (SYBR Green, TIANGEN BIOTECH) was applied to analyse the levels of mRNA or miRNA. The miDETECT A Track™ miRNA qPCR Primers specific for miRNA and U6 were both designed by RIBOBIO (Guangzhou, China). The qRT‐PCR primers were as follows:

PIK3CA forward, 5′‐CGGTGACTGTGTGGGACTTATTG‐3′ and reverse, 5′‐TGATGTAGTGTGTGGCTGTTGAAC‐3′; BCL6 forward, 5′‐CGTGAGGTGGTGGAGAACAAC‐3′ and reverse, 5′‐GGAGAAGAGGAGGCTGCTGAC‐3′; TGFB3 forward, 5′‐TTGCCACGGTCAGCCTCTC‐3′ and reverse, 5′‐GCTTCCACCCTCTTCTTCTTGATG‐3′; STAT3 forward, 5′‐ACCAAGCGAGGACTGAGCATC‐3′ and reverse, 5′‐CAGCCAGACCCAGAAGAAGAAG‐3′; PTEN forward, 5′‐TGACCAATGGCTAAGTGAAGATGAC‐3′ and reverse, 5′‐CATTACACCAGTTCGTCCCTTTCC‐3′; GAPDH forward, 5′‐ACAGCCTCAAGATCATCAGC‐3′ and reverse, 5′‐GGTCATGAGTCCTTCCACGAT‐3′. RT‐qPCR results were figured by the 2^−ΔΔCt^ method and GAPDH or U6 was served as an internal control.

### 2.6 Western blot

Total protein was extracted by Bicinchoninic Acid Protein Assay Kit (Thermo Fisher Scientific). Protein samples were separated by electrophoresis on 10% sodium dodecyl sulphate‐polyacrylamide gel electrophoresis (SDS‐PAGE) and then transferred onto polyvinylidene fluoride (PVDF) membranes (Millipore, Billerica, MA, USA). Subsequently, the membranes were incubated with primary antibodies at 4°C overnight, and then incubated for 2 h with HRP‐linked secondary antibodies. The relative expression of certain protein was measured and β‐actin was regarded as a loading control. Antibody of Caspase‐3 (ab32351), BAX (ab182733), BCL6 (ab241549) and p‐Tau (ab151559) were purchased from Abcam (Cambridge, MA, USA). Protein bands were imaged by enhanced chemiluminescence reagent (Bio‐Rad) and analysed by ImageJ software (National Institutes of Health, Bethesda, MA, USA).

### 2.7 Dual‐luciferase reporter assay

The wild type (Wt) or mutant (Mut) of BCL6 3′‐UTR containing the binding site of miR‐6076 was cloned into the pGL3‐control luciferase reporter vectors (Promega, Madison, WI, USA) for the construction of the luciferase reporter vectors. Then, the luciferase reporter vectors were co‐transfected with miR‐6076 or control mimic by using Lipofectamine 3000 (Invitrogen). At 72 h after transfection, the luciferase activities were measured with Dual‐Glo® Luciferase Assay System (Promega) and the Renilla luciferase was detected for normalization.

### 2.8 Statistical analysis

The data in the study included at least three independent samples. Statistical analysis of the data was conducted by GraphPad Prism 9.0. The differences between two or among more groups in the present study were determined using two tailed paired Student's *t*‐test or one‐way analysis of variance followed by Tukey post hoc test, respectively. Data in this study are exhibited as mean ± standard deviation. A value was considered statistically significant if *p* < 0.05.

## 3 RESULTS

### 3.1 A model of β‐amyloid‐induced neuronal injury

**FIGURE 1:**
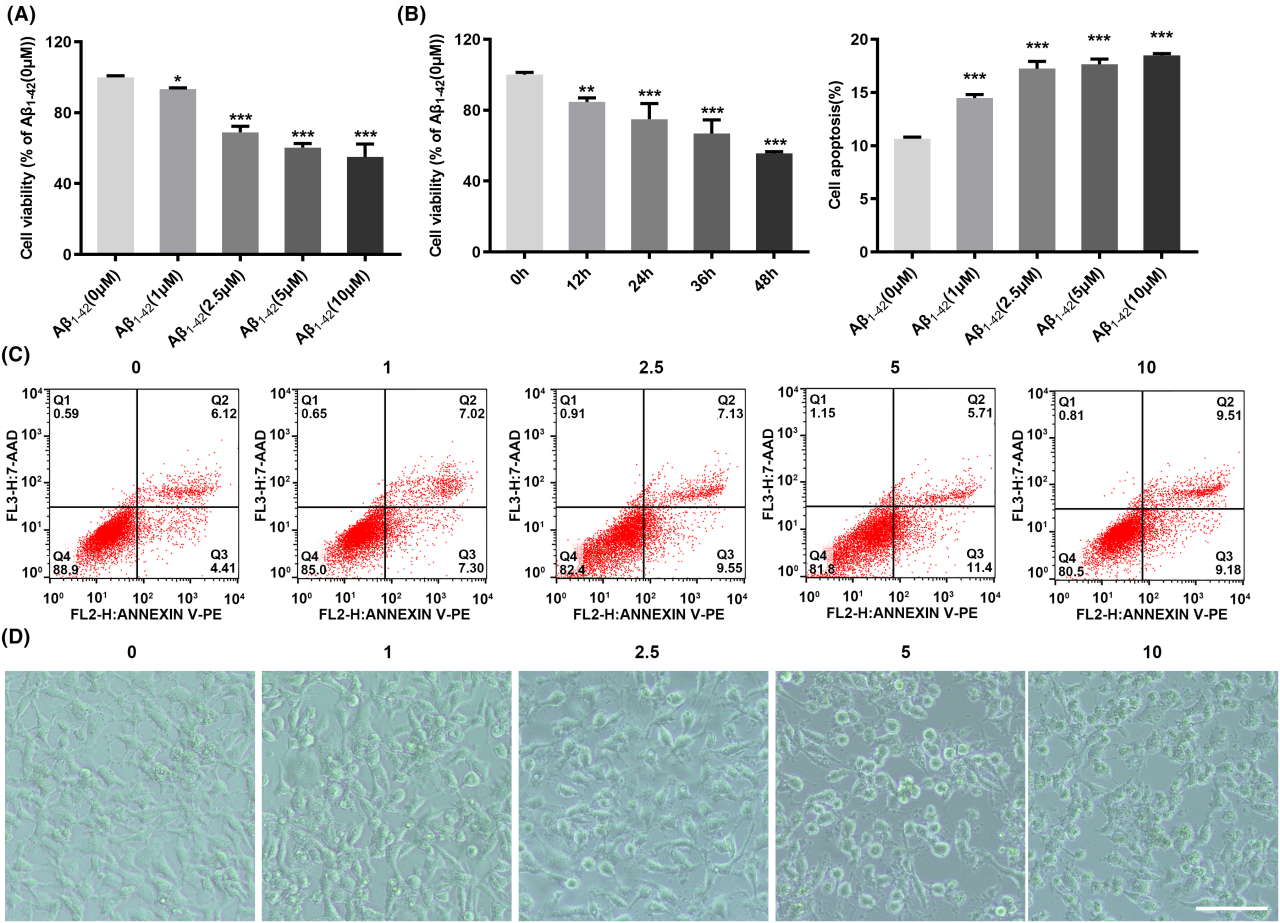
A model of β‐amyloid‐induced neuronal injury. (A, B) The viability of SH‐SY5Y cells treated with Aβ_1‐42_ was detected by CCK8 assay (C) The apoptosis of Aβ_1‐42_‐treated cells was detected by flow cytometry. (D) The morphological changes in SH‐SY5Y cells induced by Aβ_1‐42_. Bar = 200 μm. *p < 0.05, **p < 0.01, ***p < 0.001.

To construct the cell model, SH‐SY5Y cells were treated with amyloid‐β_1‐42_ (Aβ_1‐42_). Cells were treated with different concentrations of Aβ_1‐42_ (0, 1, 2.5, 5, 10 μM) for 24 h or Aβ_1‐42_ (10 μM) at different times (0, 12, 24, 36, 48 h). CCK8 assay revealed that Aβ_1‐42_ induced a dose‐ and time‐dependent decrease in the viability of SH‐SY5Y cells (Figure 1A,B). Next, we assessed the effect of different doses of Aβ_1‐42_ on apoptosis of SH‐SY5Y cells by flow cytometry analysis. Results showed that Aβ_1‐42_ induced a dose‐dependent increase ratio of apoptotic cells (Figure 1C). As shown in Figure 1D, a dose‐dependent morphological changes caused by Aβ_1‐42_, including unhealthy cell bodies and disrupted cell extensions.

### 3.2 Effects of miR‐6076 on Aβ_1‐42_‐induced cell viability and apoptosis in SH‐SY5Y cells

**FIGURE 2:**
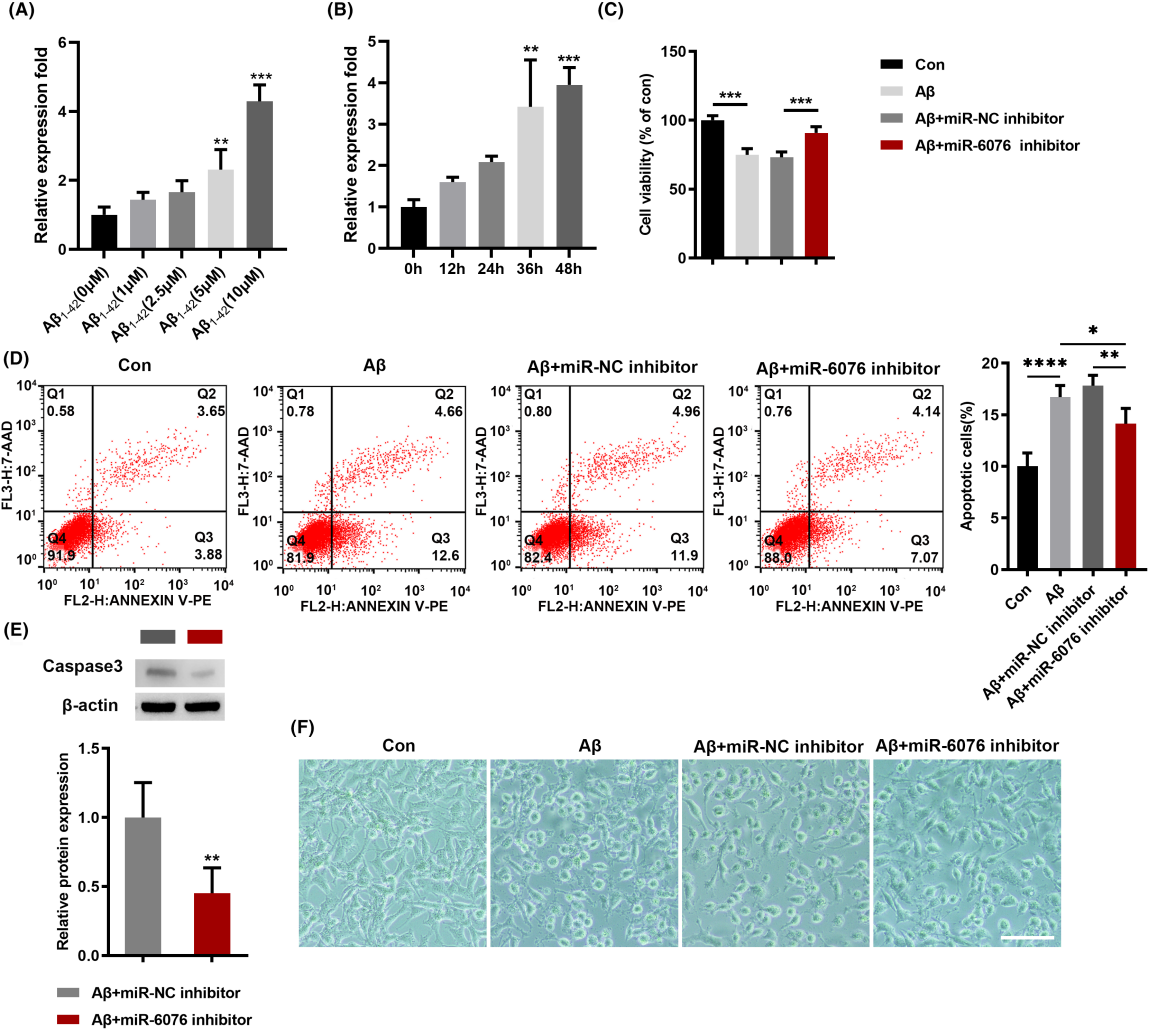
miR‐6076 inhibition attenuates Aβ_1‐42_‐induced neuronal damage. (A, B) The expression of miR‐6076 in SH‐SY5Y cells treated with Aβ_1‐42_ was detected by qRT‐PCR. (C) The viability of SH‐SY5Y cells treated with Aβ_1‐42_ was detected by CCK8 assay (D) The apoptosis of Aβ_1‐42_‐treated cells was detected by flow cytometry. (E) The expression of Caspase‐3 proteins was examined with western blot. (F) The morphological changes in SH‐SY5Y cells treated with miR‐6076 mimic. Bar = 200 μm. *p < 0.05, **p < 0.01, ***p < 0.001, ****p < 0.0001.

We assessed the expression of miR‐6076 in SH‐SY5Y cells with different concentrations of Aβ_1‐42_. The results showed that miR‐6076 was significantly increased (Figure 2A). Subsequently, the expression of miR‐6076 in SH‐SY5Y cells treated with Aβ_1‐42_ (10 μM) for different times. Similarly, the expression of miR‐6076 was obviously upregulated in a time‐dependent manner (Figure 2B). Next, CCK8 analysis showed that miR‐6076 inhibition significantly increased cell viability (Figure 2C). Flow cytometry assay showed that the number of apoptotic cells was up‐regulated by Aβ_1‐42_, while miR‐6076 inhibition partially decreased apoptosis of SH‐SY5Y cells mediated by Aβ_1‐42_ (Figure 2D). Consistent with these results, western blot analyses showed that transfection with miR‐6076 inhibitor significantly decreased the protein expression levels of Caspase‐3 (Figure 2E). As shown in Figure 2F, miR‐6076 inhibitor partially restored the morphological changes caused by Aβ_1‐42._ Taken together, miR‐6076 down‐expression could recover the viability and apoptosis of SH‐SY5Y cells induced by Aβ_1‐42_.

### 3.3 BCL6 is the direct target of miR‐6076

**FIGURE 3:**
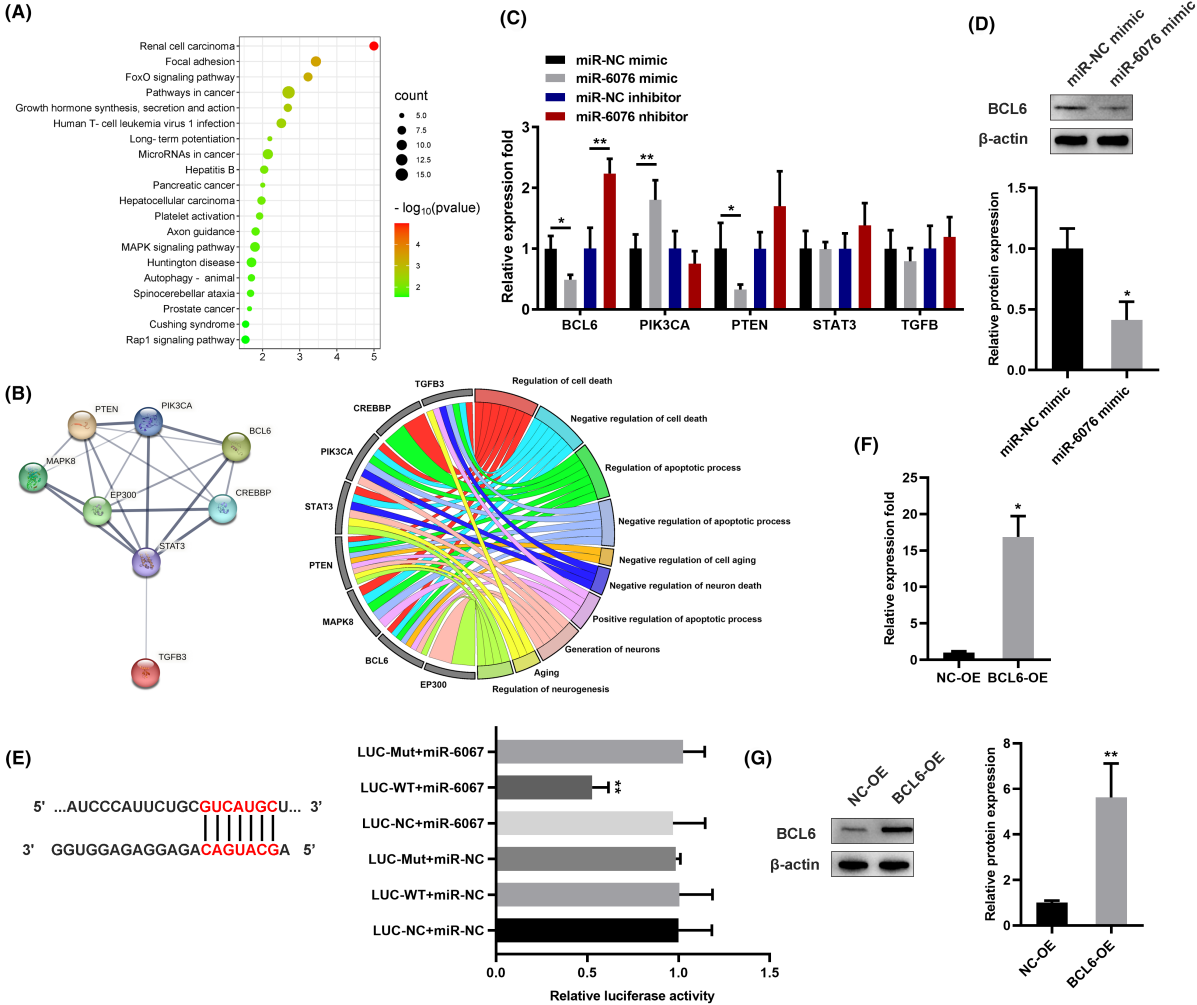
BCL6 is the direct target of miR‐6076. (A) Pathway analyses of predicted target genes of miR‐6076. (B) GO analyses of predicted target genes of miR‐6076. (C, D) qRT‐PCR and western blot analysis of predicted target genes. (E) The relative luciferase activities of SH‐SY5Y cells which were co‐transfected with miR‐6076 mimic, or NC and BCL6‐WT, or BCL6‐Mut luciferase reporter vectors. (F, G) BCL6 overexpression by plasmid in SH‐SY5Y cells. qRT‐PCR and western blot assays after transfection. *p < 0.05, **p < 0.01, ***p < 0.001.

To identify miR‐6076‐mediated downstream regulators in SH‐SY5Y cells, two target prediction algorithms (Targetscan and miRDB)^16^, ^17^ were applied. The 212 potential target genes were further analysed according to the DAVID Bioinformatics Resources.^18^ From the result, we can see that some target genes were enriched in FoxO signalling pathway (Figure 3A), which mediates cellular functions of proliferation, apoptosis and cell growth.^19^ Further analysis of these gene enriched in FoxO signalling pathway through SRTING database, the results revealed that these genes can interact with each other, and were enriched in biological processes such as apoptosis, growth and nerve regeneration. Among them, target scores greater than 90 are selected according to Targetscan for verification, including PIK3CA, BCL6, TGFB3, STAT3 and PTEN. qRT‐PCR confirmed that only BCL6 mRNA was downregulated after miR‐6076 overexpression, while upregulated after miR‐6076 inhibition (Figure 3C). Western blot confirmed that BCL6 protein levels were significantly downregulated after miR‐6076 overexpression (Figure 3D). luciferase reporter analysis was performed to determine whether BCL6 is a direct target of miR‐6076. Expression of miR‐6076 significantly decreased luciferase activity in the wild‐type BCL6, but not in the mutant form in the results of luciferase reporter (Figures 3E). Taken together, these results indicate that BCL6 is the target of miR‐6076. To determine the contribution of the BCL6 on Aβ_1‐42_‐induced cell viability and apoptosis, we upregulated the endogenous expression of BCL6 (Figure 3F,G).

### 3.4 Effects of BCL6 on Aβ_1‐42_‐induced cell viability and apoptosis in SH‐SY5Y cells

**FIGURE 4:**
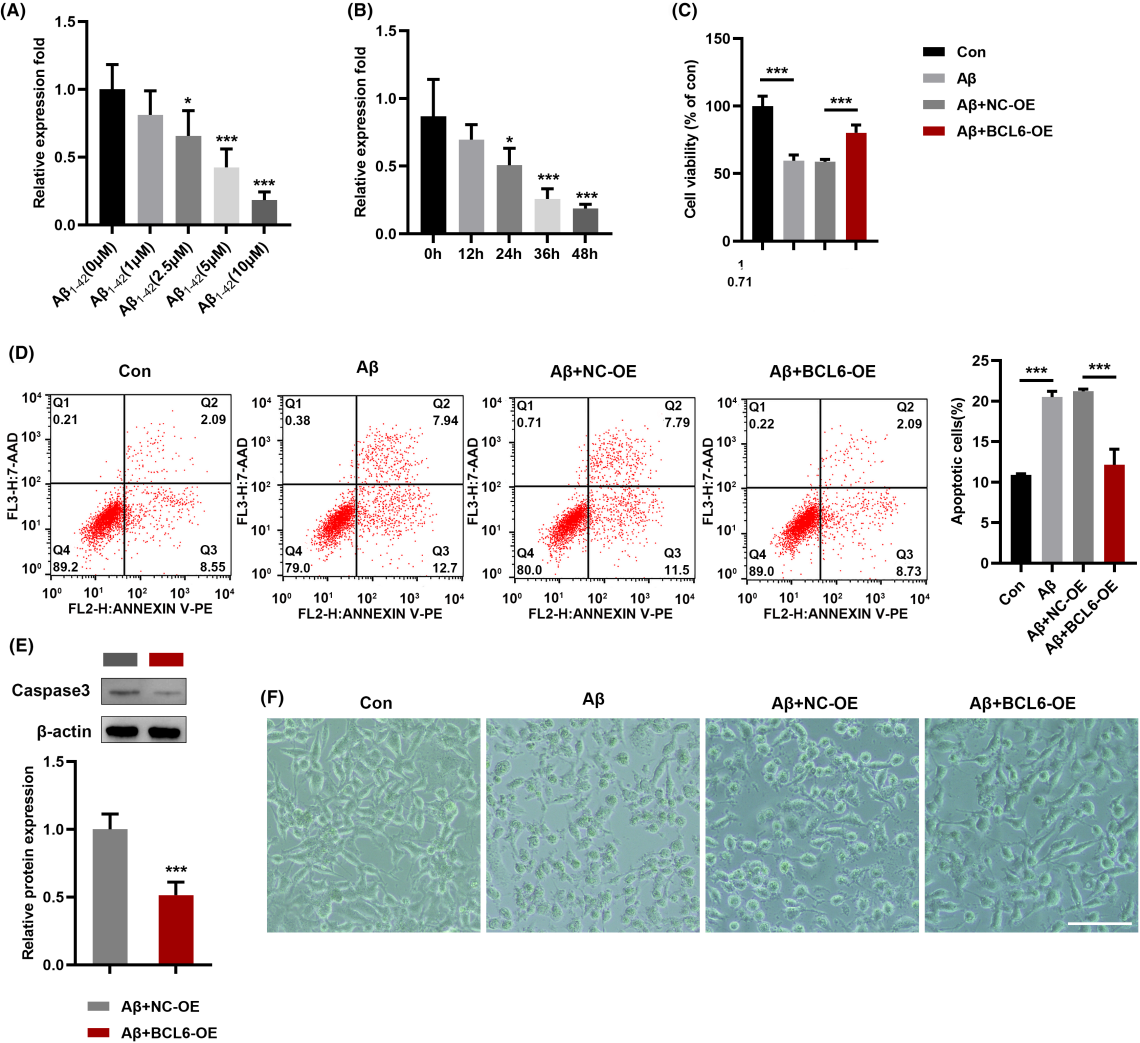
BCL6 overexpression attenuates Aβ_1‐42_‐induced neuronal damage. (A, B) The expression of BCL6 in SH‐SY5Y cells treated with Aβ_1‐42_ was detected by qRT‐PCR. (C) The viability of SH‐SY5Y cells treated with Aβ_1‐42_ was detected by CCK8 assay (D) The apoptosis of Aβ_1‐42_‐treated cells was detected by flow cytometry. (E) The expression of Caspase‐3 proteins was examined with western blot. (F) The morphological changes in SH‐SY5Y cells treated with BCL6. Bar = 200 μm. *p < 0.05, **p < 0.01, ***p < 0.001.

We assessed the expression of BCL6 in SH‐SY5Y cells with different concentrations of Aβ_1‐42_. The results showed that BCL6 was significantly decreased (Figure 4A). Subsequently, the expression of BCL6 in SH‐SY5Y cells treated with Aβ_1‐42_ (10 μM) for different times. Similarly, the expression of BCL6 was obviously downregulated in a time‐dependent manner (Figure 4B). Next, CCK8 analysis showed that BCL6 overexpression significantly increased cell viability (Figure 4C). Flow cytometry assay showed BCL6 overexpression partially decreased apoptosis of SH‐SY5Y cells mediated by Aβ_1‐42_ (Figure 4D). Consistent with these results, western blot analyses showed that transfection with BCL6 significantly decreased the protein expression levels of Caspase‐3 (Figure 4E). As shown in Figure 4F, BCL6 overexpression partially restored the morphological changes caused by Aβ_1‐42._ Taken together, BCL6 overexpression could recover the viability and apoptosis of SH‐SY5Y cells induced by Aβ_1‐42_.

### 3.5 miR‐6076 could reverse the effects of BCL6 in SH‐SY5Y cells

**FIGURE 5:**
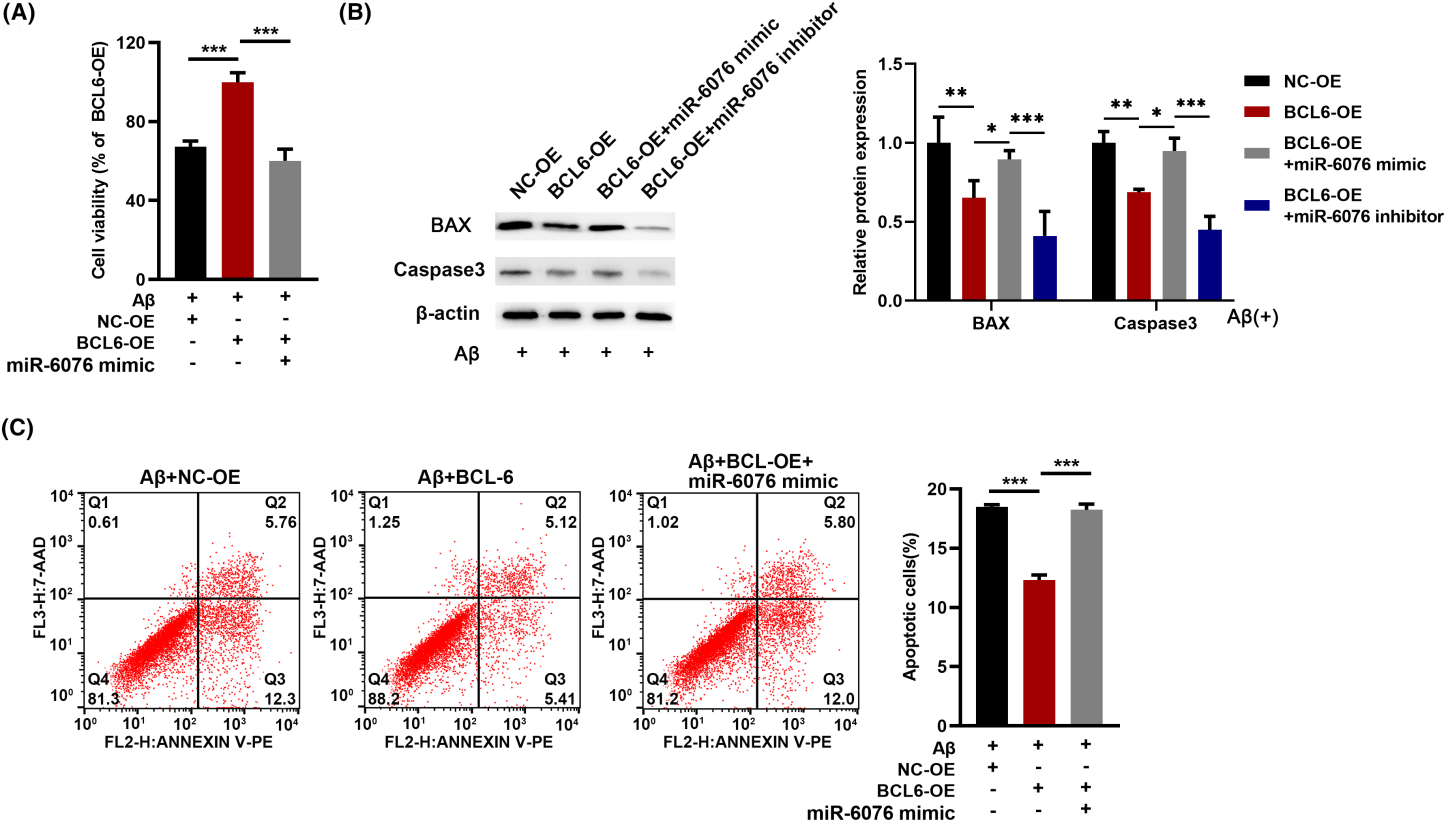
miR‐6076 could reverse the effects of BCL6 in SH‐SY5Y cells. (A) Aβ_1‐42_‐treated SH‐SY5Y cells were transfected with BCL6 or miR‐6076 inhibitor. The viability of SH‐SY5Y cells was detected by CCK8 assay. (B) The expression of BAX, Caspase‐3 was detected by western blot. (C) The apoptosis of Aβ_1‐42_‐treated cells was detected by flow cytometry. *p < 0.05, **p < 0.01, ***p < 0.001.

To further investigate the functional interaction between miR‐6076 and BCL6 in SH‐SY5Y cells, several rescue experiments were carried out by co‐transfection of miR‐6076 mimic or BCL6 overexpression vector. As shown in Figure 5A,C, BCL6 overexpression could promote cell survival and reduce apoptosis in Aβ_1‐42_‐treated SH‐SY5Y cells, while this increase was inhibited by miR‐6076 overexpression. In addition, miR‐6076 inhibition further promoted cell survival and reduce cell apoptosis (Figure 5B). Collectively, these experiments demonstrated that miR‐6076 could regulate Aβ_1‐42_‐induced cell viability and apoptosis by targeting BCL6.

### 3.6 Effects of miR‐6076‐BCL6 mediated the levels of p‐Tau of SH‐SY5Y cells

**FIGURE 6:**
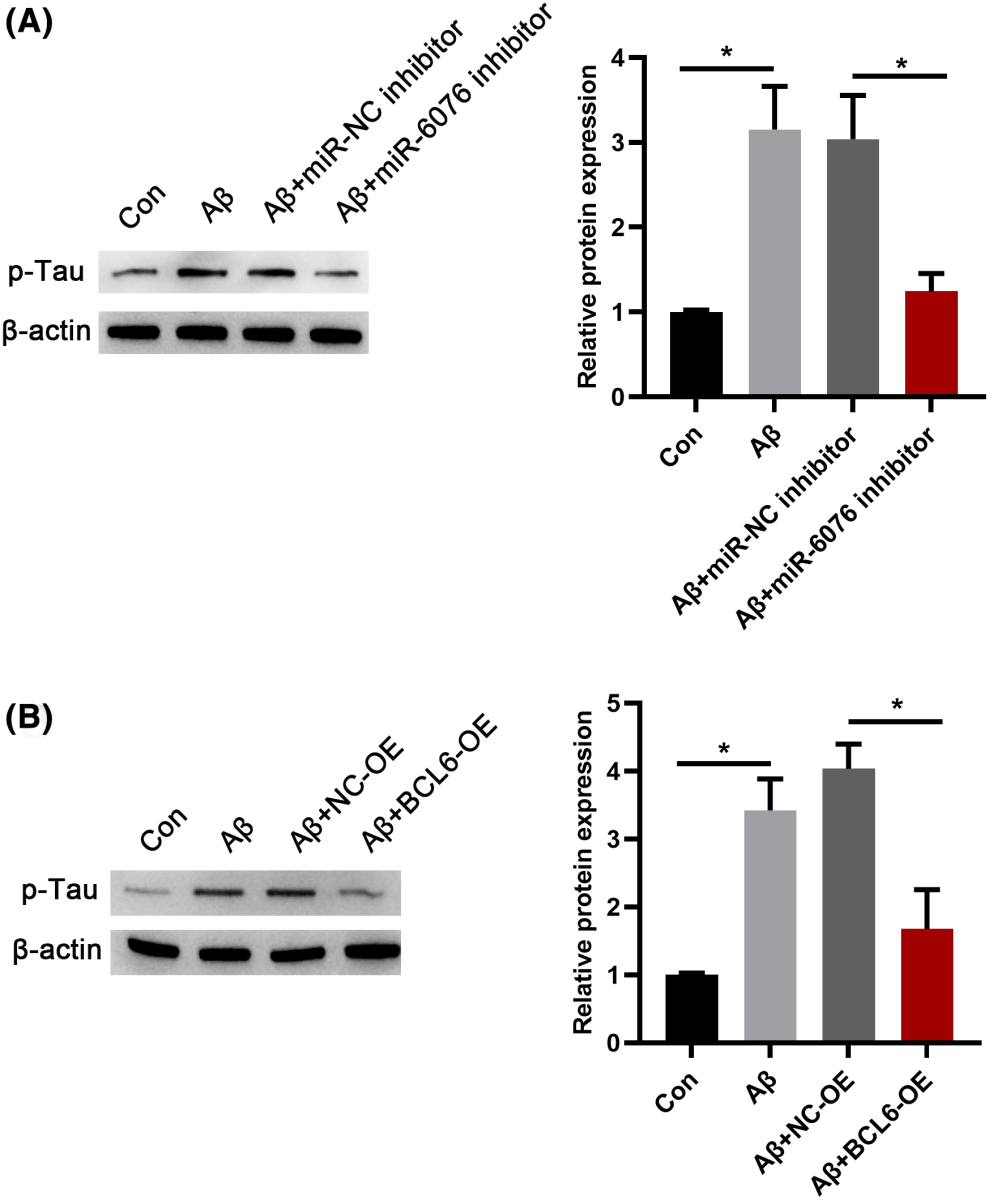
Effects of miR‐6076‐BCL6 mediated the levels of p‐Tau of SH‐SY5Y cells. (A, B) Western blot analysis was performed to examine the levels of p‐Tau SH‐SY5Y cells with or without Aβ_1‐42_ treatment were transfected with BCL6 or miR‐6076 inhibitor. *p < 0.05.

Neurofibrillary tangles are composed of highly p‐Tau protein, and its accumulation is deemed to be closely connected to cognitive recession of AD. Previous studies have shown that p‐Tau was upregulated in mice via intraventricular injection of Aβ_1‐42._ ^20^, ^21^ Here, the results of the present study showed that SH‐SY5Y cells exposed to Aβ_1–42_ could induce hyperphosphorylation of tau. We further probed whether miR‐6076 affects the levels of p‐Tau in Aβ_1‐42_‐treated SH‐SY5Y cells. Western blot analysis manifested that miR‐6076 inhibitor could reduce the level of p‐Tau in cells (Figure 6A). Similarly, the relative expression level neuronal markers were upregulated, and p‐Tau level was decreased by BCL6 overexpression (Figure 6B). These results indicated that miR‐6076 promoted the Aβ1–42‐induced hyperphosphorylation of tau in SH‐SY5Y cells, while BCL6 suppressed the Aβ1–42‐induced hyperphosphorylation of tau.

## 4 DISCUSSION

Aβ can self‐aggregate to form oligomers which are toxic to cause membrane defects, disruption to neuronal networks, neuronal dysfunction and changes in animal behaviour.^22^, ^23^, ^24^ Studies has shown that the mean level of Aβ_1–42_ in the cerebrospinal fluid (CSF) are significantly reduced in subjects with AD relative to age‐matched controls.^25^ Thus, Aβ_1‐42_ measurement in CSF is an important biochemical marker for differentiating AD from non‐AD dementia. The results of the present have shown that Aβ_1‐42_ peptide decreases neural viability, increases apoptosis and mitochondrial biogenesis in neurons,^26^ which is similar to the pathology of AD, and was widely used to construct AD model in vitro. Here, we constructed the β‐amyloid‐induced cell model, and found that Aβ could repressed viability and promoted apoptosis of SH‐SY5Y cells.

Using DNA arrays, miRNA arrays, RNA‐sequencing, Northern dot blot hybridization technologies, ELISA, western immunoblot and bioinformatics analysis, many laboratories have independently analysed miRNA abundance, speciation and complexity in AD brain compared to age‐matched controls.^27^, ^28^ For example, miR‐200b/c is proved to reduce the secretion of toxic Aβ_1‐42_ and prevent memory loss and learning disabilities.^29^ Similarly, upregulated miR‐195 and miR‐124 were able to decrease BACE1 expression and Aβ levels in AD cellular models.^30^ In the present study, miR‐6076 was upregulated in Aβ1‐42‐treated SH‐SY5Y cells. Moreover, miR‐6076 inhibition could restore Aβ‐mediated viability, and apoptosis of SH‐SY5Y cells. These findings manifested that miR‐6076 acted as a pathogenic factor in AD.

miRNA is a subset of small non‐coding RNAs that regulates gene expression at posttranscriptional level.^31^ BCL6 was identified to be a target of miR‐6076. A review of previous studies found that BCL6 is involved in neurogenesis. For example, Wiegreffe et al.^32^ found BCL6 was involved in the transition of cortical neurons from progenitor to postmitotic differentiation state. Deletion of Bcl6 in cortical projection neurons induces pronounced cell death. Wei et al.^33^ found that BCL6 inhibition may attenuate ODG‐induced neuronal damage. In addition, cortical neurogenesis requires Bcl6‐mediated transcriptional repression of most signalling pathways promoting cortical progenitor self‐renewal.^34^ In our study, Aβ_1‐42_ treatment inhibited BCL6 expression, and BCL6 overexpression suppressed cell apoptosis and improved cell viability.

The key pathological hallmarks‐extracellular plaques and intracellular neurofibrillary tangles (NFT), which are caused by tau hyperphosphorylation are central to the post‐mortem diagnosis of AD.^35^ The coexistence of Aβ plaques and Tau neurofibrillary tangles is linked to neural system failure and cognitive decline in AD. Research indicates that accumulated Aβ may induce the hyperphosphorylation of Tau, and Aβ toxicity is critically dependent on the presence of Tau.^36^, ^37^ The results of the present study showed that SH‐SY5Y cells exposed to Aβ_1–42_ could induce hyperphosphorylation of tau. We further probed whether miR‐6076 or BCL6 affects the levels of p‐Tau in Aβ_1‐42‐_treated SH‐SY5Y cells. The data showed that interference of miR‐6076 or BCL6 overexpression inhibited the levels of p‐Tau in Aβ_1‐42_‐treated SH‐SY5Y cells. From the findings of the above studies, together with the findings of the present study, it may be possible to propose that miR‐6076 regulated Aβ‐induced hyperphosphorylation of tau by targeting BCL6. This study had several limitations, and was a preliminary study that included the therapeutic effects of miR‐6076 or BCL6 in AD, as well as the pathways that may be involved.

In conclusion, our findings demonstrated that miR‐6076 regulates neuronal survival through the intrinsic apoptosis pathway by targeting BCL6. Inhibition of miR‐6076 or overexpression of BCL6 could attenuate Aβ_1‐42_ mediated p‐Tau level. Therefore, we concluded that miR‐6076/ BCL6 axis may serve as a potential diagnostic and therapeutic target for Aβ_1‐42_‐induced neuronal damage.

## AUTHOR CONTRIBUTIONS

**Yujian Lin:** Conceptualization (equal); data curation (equal); formal analysis (equal); funding acquisition (equal); investigation (equal); methodology (equal); project administration (equal); resources (equal); software (equal); supervision (equal); validation (equal); visualization (equal); writing – original draft (equal); writing – review and editing (equal). **Lei Zhang:** Data curation (equal); formal analysis (equal); funding acquisition (equal); investigation (equal); methodology (equal); project administration (equal); resources (equal); software (equal); supervision (equal); validation (equal); visualization (equal); writing – original draft (equal). **Mengyue Gao:** Data curation (equal); formal analysis (equal); investigation (equal); methodology (equal); supervision (equal); validation (equal); visualization (equal). **Zixin Tang:** Data curation (equal). **Xiang Cheng:** Funding acquisition (equal); investigation (equal); methodology (equal); supervision (equal); validation (equal); visualization (equal). **Haoming Li:** Investigation (equal); methodology (equal); supervision (equal); validation (equal); visualization (equal). **Jianbing Qin:** Investigation (equal); methodology (equal); supervision (equal); validation (equal); visualization (equal). **Meiling Tian:** Investigation (equal); methodology (equal); validation (equal); visualization (equal). **Guohua Jin:** Funding acquisition (equal); investigation (equal); methodology (equal); project administration (equal); supervision (equal); validation (equal); visualization (equal). **Xinhua Zhang:** Conceptualization (equal); data curation (equal); investigation (equal); methodology (equal); supervision (equal); validation (equal); visualization (equal). **Wen Li:** Conceptualization (equal); data curation (equal); formal analysis (equal); funding acquisition (equal); investigation (equal); methodology (equal); project administration (equal); resources (equal); software (equal); supervision (equal); validation (equal); visualization (equal); writing – original draft (equal); writing – review and editing (equal).

## FUNDING INFORMATION

Contract grant sponsor: Graduate Scientific Research Innovation Program of Jiangsu Province; Contract grant number: KYCX19 2066. Contract grant sponsor: National Natural Science Foundation of China; Contract grant number: 31171038. Contract grant sponsor: Jiangsu Natural Science Foundation; Contract grant number: BK2011385. Contract grant sponsor: Jiangsu “333” program funding; Contract grant number: BRA2016450. Contract grant sponsor: Application Research Project of Nantong City; Contract grant number: MS12017015‐3. Contract grant sponsor: The Training Program of Innovation and Entrepreneurship for Graduates of Nantong University of China; Contract grant number: No. 265. Contract grant sponsor: a Project Funded by the Priority Academic Program Development (PAPD) of Jiangsu Higher Education institutions. Contract grant number: 03081023 (Not applicable). Contract grant sponsor: Nantong Science and Technology Project; Contract grant number: JC2021056.

## CONFLICT OF INTEREST STATEMENT

The authors have no conflict of interest to declare.

## Data Availability

The data that support the findings of this study are available on request from the corresponding author.
